# EBV-Positive Gastric Cancer: Current Knowledge and Future Perspectives

**DOI:** 10.3389/fonc.2020.583463

**Published:** 2020-12-14

**Authors:** Keran Sun, Keqi Jia, Huifang Lv, Sai-Qi Wang, Yan Wu, Huijun Lei, Xiaobing Chen

**Affiliations:** ^1^ Department of Oncology, The Affiliated Cancer Hospital of Zhengzhou University, Henan Cancer Hospital, Zhengzhou, China; ^2^ Department of Pathology, Pathology Department of Hebei Medical University, Shijiazhuang, China

**Keywords:** Epstein–Barr virus, gastric cancer, miRNA, DNA methylation, immune checkpoint

## Abstract

Gastric cancer is the fifth most common malignant tumor and second leading cause of cancer-related deaths worldwide. With the improved understanding of gastric cancer, a subset of gastric cancer patients infected with Epstein–Barr virus (EBV) has been identified. EBV-positive gastric cancer is a type of tumor with unique genomic aberrations, significant clinicopathological features, and a good prognosis. After EBV infects the human body, it first enters an incubation period in which the virus integrates its DNA into the host and expresses the latent protein and then affects DNA methylation through miRNA under the action of the latent protein, which leads to the occurrence of EBV-positive gastric cancer. With recent developments in immunotherapy, better treatment of EBV-positive gastric cancer patients appears achievable. Moreover, studies show that treatment with immunotherapy has a high effective rate in patients with EBV-positive gastric cancer. This review summarizes the research status of EBV-positive gastric cancer in recent years and indicates areas for improvement of clinical practice.

## Introduction

Epstein–Barr virus (EBV) is the main pathogenic factor for nasopharyngeal carcinoma. However, studies find that EBV infection is also associated with the development of T-cell lymphoma and EBV-associated gastric cancer (1, 2). In 1990, Burke et al. (3) detected EBV in gastric lymphoepithelial carcinoma, and this was the first report on histopathological features of EBV-positive gastric cancer. Furthermore, Shibata and Weiss (4) find EBV to be associated with gastric adenocarcinoma. They demonstrate the presence of the EBV genome specifically in gastric cancer cells and adjacent dysplastic epithelium, but not in surrounding normal cells. Studies find EBV-positive and -negative gastric cancer to have different pathogens and that EBV may play an important role in its pathogenesis (5, 6). In 2004, van Beek et al. (7) analyzed the clinicopathological features of EBV-positive and -negative gastric adenocarcinomas. The results show that EBV-positive gastric cancer has a unique genomic aberration, obvious clinicopathological features, and good prognosis. In 2009, Murphy et al. (8) found EBV-positive gastric cancer to be different from other gastric cancers in terms of patients’ sex, tumor anatomical site, and surgical anatomical structure. In 2014, Liang et al. (9) studied the mechanism of occurrence and development of EBV-positive gastric cancer. The comprehensive epigenomic and transcriptomic analysis identified 216 genes downregulated by EBV-induced hypermethylation; in EBV-positive tumors, the methylation of *ACSS1*, *FAM3B*, *IHH*, and *TRABD* was significantly increased. Moreover, five signaling pathways (axon guidance, local adhesion formation, interaction between cytokines and receptors, mitogen-activated protein kinase signal transduction, and actin cytoskeleton regulation) were significantly affected by EBV-related genomic and epigenomic changes. However, no specific treatment has been found for EBV-positive gastric cancer, and the EBV-titer is not associated with the risk of gastric cancer (10, 11). Several recent studies find a close relationship between EBV-positive gastric cancer and immune checkpoints (12). In 2018, Panda et al. (13) found EBV-positive gastric cancer with low mutation burden to be a subset of microsatellitestable (MSS) gastric cancer, which may respond to immune checkpoint therapy. Thus, EBV-positive gastric cancer is now considered a unique molecular subtype of gastric cancer (14) and is associated with good prognosis in patients (15). At present, there is no article to summarize and analyze the characteristics, mechanisms, and treatment of EBV-positive gastric cancer, including latency proteins. MicroRNAs (miRNAs) and DNA methylation have important effects on EBV-positive gastric cancer; there is a close relationship between them, and it may reveal potential treatments for EBV-positive gastric cancer. Here, we review recent advances in EBV-positive gastric cancer research to improve the current understanding of this disease and aid in development of newer treatment modalities for this cancer type.

## Characteristics of EBV-Positive Gastric Cancer

Gastric cancer is normally classified on the basis of its histomorphological characteristics (16). The Cancer Genome Atlas reports a comprehensive identification of genetic changes associated with gastric cancer and further divides this form of cancer into four subtypes: EBV-positive tumors (9%), microsatellite unstable tumors (22%), genetically stable tumors (20%), and chromosome unstable tumors (50%). Moreover, EBV-positive and MSI gastric cancers have the capability to respond to newer immunotherapy drugs (17). However, as opposed to general gastric cancer, EBV-positive gastric cancer, despite having unique pathological characteristics, has no specific clinical manifestations. A few studies have found higher incidences of EBV-positive gastric cancer in men and patients below the age of 60 years. Camargo et al. (18) find that the average age of EBV-positive gastric cancer patients is 58 years old, and 71% of them are men. EBV-positive gastric cancer often occurs in the proximal stomach (cardia and gastric body), where it forms lumps or ulcers that are accompanied by lymphocyte infiltration. Another noteworthy feature of EBV-positive gastric cancer is the ease of invasion into the submucosa with a low rate of lymph node metastasis. A majority of patients were diagnosed in the advanced stage (52%, stage III and IV), and 2247 (49%) patients died during the median follow-up period of 3 years. An unadjusted Cox regression analysis indicates that the median survival duration of EBV-positive gastric cancer patients is 8.5 years although that of EBV-negative patients is only 5.3 years. It is evident that the prognosis and effective treatment rate of gastric cancer patients with positive EBV is more desirable. We studied the proper treatment to prolong the survival time in EBV-positive gastric cancer patients considering the curable nature of EBV-positive gastric cancer.

## How to Test EBV-Positive Gastric Cancer

### Immunohistochemistry and In Situ Hybridization

The principles of immunohistochemistry and in situ hybridization (ISH) are different, and detection results vary. The EBER-1 probe used in ISH is a base sequence that can specifically anneal to the small mRNA encoded by the EBV. The probe can detect gastric cancer specimens fixed by formaldehyde and embedded in paraffin, enabling detection of EBV in tumor cells in situ with accurate localization and strong specificity. However, the gastric cancer tissue is often selected to avoid wasting reagents as the EBER-1 probe is expensive. Occasionally the fixation of gastric cancer tissue is poor, the nucleic acid in the tissue is denatured and diffused, the effective binding sites are reduced, and the staining may appear as weak positive or false negative markers. Immunohistochemical detection is based on the LMP-1 membrane protein encoded by EBV, which cannot detect the location or transcriptional quantity of the virus. However, compared with ISH, immunohistochemical methods have the advantage of simple steps, convenient operation, high sensitivity, and low price, making it a reliable primary screening method for EBV. Immunohistochemical positives can be followed up by ISH to exclude the possibility of false positives. The combination of the two methods might improve the accuracy of detection by reducing the chances of false positives and negatives.

### Genome Sequencing

EBV-positive gastric cancer is traditionally identified by ISH of viral nucleic acid (19). However, genome sequencing is a potential alternative. Camargo et al. (20) determine the normalized EBV readings in 295 fresh gastric cancer samples by whole genome, whole exome, mRNA, and miRNA sequencing. Formalin-fixed, paraffin-embedded tissue sections were obtained and used for ISH confirmation in 13 cases with high EB viral load and 11 cases with low EBV. In pairwise comparisons, individual samples are uniformly either high or low in all genomic methods for which data are available. The empirical cutoff value of sequencing count confirmed 26 (9%) tumors to be EBV-positive. EBV was either positive or negative based on molecular detection. Conversely, the Epstein–Barr encoding region (EBER)-ISH was either positive or negative in all samples except for one, which was evaluated by the two methods (kappa=0.91). Thus, EBV-positive gastric tumors can be accurately identified by quantifying virus sequences in genomic data. Moreover, simultaneous analysis of human and viral DNA, mRNA, and miRNA can simplify the tumor profile of clinical nursing and research.

### Detection of Anti-EBV and Anti-p53 Antibodies

Tumor protein p53, or simply p53, is closely related to the occurrence of gastric cancer, and many studies find EBV infection to be associated with p53 methylation (21–23). In 2019, Camargo et al. found that EBV-positive gastric cancer cases lack the *TP53* mutation, suggesting that serological characteristics may provide information for viral carcinogenesis. Consistent with the prevalence of EBV, 99% of patients tested positive for the anti-Epstein–Barr virus nuclear antigen 1 (EBNA) antibody, and 98% of the patients tested positive for the antiviral capsid antigen antibody regardless of the EBV status of the tumor. The levels of p53 antibody and EBV-positivity were negatively correlated. The positive rate of anti-p53 staining was 15% in the literature. However, the results suggest dissimilar correlations between anti-EBV antibody, anti-p53 antibody and tumor EBV-positivity.

### Droplet Digital PCR

Droplet digital PCR (ddPCR) is the latest method that can be used in the detection of *Plasmodium falciparum* (24), multiple viruses (25), nervous system lymphomas (26), etc. A ddPCR-based screening method for detection of EBV–associated gastric carcinoma was established in 2019 (27, 28). This method uses the ddPCR method to calculate EBV-DNA load according to the copy number of EBV BamH1-W fragments and sets the cutoff value of the EBV-DNA load.

## What Is the Mechanism of Occurrence and Development of EBV-Positive GC?

Epstein and Barr discovered the EBV in 1964 and identified its original host as the human body; the virus had the capability to infect B-lymphocytes, epithelial cells, and fibroblasts (29, 30). EBV infection in the human body does not immediately lead to gastric cancer. Although the infection rate of EBV in adults is 90%, the incidence rate of gastric cancer remains low; the majority of individuals only carry the virus during the incubation period (31). At present, there are two theories about the mechanisms of EBV infection. The first is that EBV infects B-lymphocytes and oral epithelial cells. As the saliva containing EBV enters the digestive tract, EBV directly infects the epithelial cells. The second theory is that EBV is reactivated in some way in B-lymphocytes in the stomach and then released to infect the epithelial cells (32). Moreover, lymphocytes infected by EBV can encounter epithelial cells through integrin β-1/β-2 and promote cell-to-cell contact by translocating intracellular adhesion molecule-1 to the cell surface. Finally, the virus particles are transmitted through the endocytosis pathway mediated by reticular proteins (33). After phagocytosis, EBV-DNA is transported to the nucleus, where the exposed linear DNA genome is assembled into a functional, small, circular chromosome. After circularization, the viral genome chromatinization can effectively protect it from DNA damage and ensure strict regulation of gene expression (34). The CpG motif of the viral genome is widely methylated, thus successfully establishing latent infection. EBV primarily infects host cells in two ways: lytic and latent infection. However, the virus mostly remains in the latent infection state without replication (19). After entering the incubation period in human bodies, the EBV prompts methylation of the host genome, imbalance of the cellular signaling pathway, abnormal gene expression, generation of a tumor microenvironment of infected gastric epithelial cells, and initiation and development of gastric cancer. Moreover, latent EBV gene products, such as EBERs, BARF-0, EBNA-1, and LMP2A, are involved in the downregulation of the miR-200 family, resulting in reduced E-cadherin expression, which is a key step in the carcinogenesis of EBV-associated gastric cancer (EBVaGC) (35).

### Virus Latency Gene Product

EBV has three types of latent phases. As EBV-positive gastric cancer is type I latency, EBERs, EBNA-1, miR-BARTS, and LMP2A are highly expressed and play an important role in viral replication (36, 37). EBER1 upregulates the expression of insulin growth factor-1, thus promoting proliferation of EBVaGC cells (38). EBERs associate with IL-6-STAT3 signaling pathway to induce chemotherapy resistance and promote cell migration (39).

EBNA-1 is an important molecule for EBV latent infection (40, 41). It binds to the viral ORIP sequence in a sequence-dependent manner and aids in EBV attachment to the host cell chromosome (42). EBNA-1 is also a transactivator of viral genes that may induce accumulation of reactive oxygen species (ROS) mediated by miR-34a and NOX2, regulating the activity of tumor cells (43). Additionally, EBNA-1 can lead to the loss of promyelocytic leukemia protein nuclear bodies in the nucleosome of promyelocytic leukemia and weaken the cellular response to DNA damage (44).

BARF-0 and BARF-1 are also involved in the latent state of EBV infection. BARF-0 downregulates the expression of TET2 (45). BARF-1 gene silencing triggers caspase-dependent mitochondrial apoptosis (46). It can also induce alteration in the NF-κB/miR-146a/Smad4 pathway and expression of cyclin-D1 protein in gastric cancer cells (47–49). Moreover, BARF-1 can activate the cell cycle regulator bcl-2 (50). These processes promote proliferation of gastric cancer cells.

LMP2A is the most important molecule in the incubation period of EBV. It can activate the NF-κB-Survivin pathway (51, 52), regulate the expression of cyclin-E and proportion of cells in the S phase (53), mediate Notch signaling, and promote mitochondrial division and cell migration (54). Additionally, it can upregulate miR-155-5p through the NF-κB pathway and inhibit the activation of Smad2 and p-Smad2 (55, 56). LMP2A can also initiate gastric cancer by upregulating or downregulating other genes. For instance, LMP2A downregulates the expression of TET2, COX-2, and HLA (45, 57, 58) and upregulates FOXO1 and FOXO3 (59). LMP2A activates the PI3K/AKT pathway to mediate the transformation process and inhibits apoptosis-induced proliferation by transforming growth factor β1 (60). LMP2A induces STAT3 phosphorylation, resulting in *DNMT1* transcriptional activation and *PTEN* promoter methylation (61). LMP2A also activates CpG island methylation of the *AQP3* promoter, induces ERK phosphorylation, and activates *DNMT3a* transcription, which results in the loss of AQP3 expression (**Table 1**) (55).

**Table 1:** Summary of EBV proteins expressed during latency period.

EBV latency gene products	Summary of findings	References
EBERs	High expression in EBV positive gastric carcinoma	(36, 37)
	upregulates the expression of insulin growth factor-1	(38)
	cooperate with IL-6-STAT3 signaling pathway	(39)
	downregulation of miR-200 family	(35)
EBNA-1	High expression in EBV-positive gastric carcinoma	(40, 41)
	Assist EBV in binding to host chromosomes	(42)
	Protect EBVDNA	(44)
	Adjust the miR34a-NOX2-ROS signal.	(43)
	Downregulation of miR-200 family	(35)
	Change the expression of p53 gene	(21)
LMP-2A	Expression in EBV-positive gastric carcinoma	(36, 37)
	Downregulation of miR-200 family	(35)
	Activates NF-κ B-Survivin pathway	(51, 52)
	Regulates the expression of cyclin E and the proportion of S phase cells	(53)
	Mediates Notch signal	(54)
	Downregulates HLA	(58)
	Activates PI3K/AKT pathway	(60)
	Upregulates miR155-5p thus inhibiting smad2 and p-smad2.	(55, 56)
	Induces STAT3 phosphorylation	(61)
	Up-regulate FOXO1 and FOXO3	(59)
	Suppresses COX-2 by reducing TRAF2	(57)
	Activates CpG island methylation of AQP3 promoter	(55)
	Downregulates the expression of TET2	(45)
BARF0	Downregulation of miR-200 family	(35)
	Downregulates the expression of TET2	(45)
BARF1	Induces NFκB/miR-146a/SMAD4 alterations	(47)
	Triggers caspase-dependent mitochondrial apoptosis	(46)
	Activates NF-κ B-Survivin pathway	(48)
	Induces the expression of CyclinD1 protein	(49)
	Activates the cell-cycle regulator bcl-2.	(50)

### MicroRNAs

EBV is a ubiquitous human carcinogenic virus and is also the first human virus to express miRNAs. The EBV genome contains two regions that encode more than 40 miRNAs that regulate the expression of viral and human genes, such as ebv-miR-BART-1-3p, -2-5p, -3-3p, -4-5p, -5-5p, -7-3p, -9-3p, -10-3p, -17-5p, -10-3p, -18-5p, BART11, etc. (62–67). Studies suggest that EBV miRNAs affect immune response and antigen presentation and recognition, alter the communication between T- and B-cells, drive the production of antibodies during infection, and play a role in apoptosis. Additionally, EBV can induce B-cell transformation and participate in the mechanism of human tumorigenesis. Although EBV infection is related to the occurrence of several diseases, the role of miRNAs remains unclear. Extensive data describes the role of EBV miRNAs in nasopharyngeal carcinoma, and several studies have attempted to evaluate their role in gastric cancer and lymphoma. Song et al. (68) find that the EBV miRNA BART11 downregulates Foxp1 transcription factor, which promotes epithelial-mesenchymal transition by directly affecting gastric tumor cells or indirectly affecting the tumor microenvironment. BART11 also accelerates tumor invasion and metastasis, affecting survival and prognosis in patients. Dong et al. (69) find that BART10-3p and BART22 activate the Wnt signaling pathway by targeting APC and Dkk1, which play an important role in promoting EBVaGC metastasis, thus providing new prognostic biomarkers and potential therapeutic targets in EBVaGC. Wang et al. (70) report that BART3-3p promotes growth and inhibits senescence of gastric cancer cells induced by oncogenes (*RAS*) or chemotherapy (irinotecan). BART3-3p inhibits the senescence of gastric cancer cells in nude mice by modifying the aging-related SP (SASP) and the infiltration of natural killer cells and macrophages in tumors. BART3-3p directly targets the inhibition of the tumor suppressor gene and leads to the downregulation of p21, the downstream target of p53. The clinical analysis of EBV-positive gastric cancer also displays a negative correlation between the expression of BART3-3p and p21. This study suggests that the expression of BART3-3p is important in the carcinogenesis of EBV-positive gastric cancer. Other miRNAs also play an important role in this process. For instance, miR-BART5 upregulates p53 with PUMA as the target, promoting the survival of host cells (71, 72); miR-BART3-5p targets DICE1 tumor suppressor, and promotes the growth and transformation of cancer cells (73); miR-BART9 specifically inhibits E-cadherin to induce a mesenchymal-like phenotype (74, 75); miR-BART9, -11, and -12 downregulates Bim expression (76); EBV-miR-BART4-5p has an antiapoptotic role that regulates Bid expression in EBV-associated gastric carcinoma (77); EBV-miR-BART20-5p regulates cell proliferation and apoptosis by targeting BAD (78); and miR-BART16 abrogates the production of IFN-stimulated genes in response to IFN-α stimulation and inhibits the antiproliferative effect of IFN-α in latently infected cells (79). Modulation of expression of LMP2A by newly identified EBV-encoded miRNAs, miR-BART22 (80) and miR-BART17-5p, promotes migration and anchorage-independent growth by targeting kruppel-like factor 2 in gastric cancer (81, 82). EBV-miR-BART15-3p targets the anti-apoptotic TAX1BP1 and NLRP3 genes in cancer cells, thus increasing apoptosis (**Table 2**) (84–86).

**Table 2:** Summary of EBV-miRNAs expressed during gastric cancer pathogenesis.

EBV related MIRNA****	Summary of findings****	References****
miR-BART5	upregulates p53	(71, 72)
miR-BART3-3p	leads to the downregulation of p21	(70)
miR-BART3-5p	targets DICE1 tumor suppressor	(73)
miR-BART9	inhibits E-cadherin to induce a mesenchymal-like phenotype	(74, 75)
miR-BART5-3p	inhibits p53 Expression,	(62)
miR-BART5-5p/BART7-3p/BART9-3p/BART14-3p	regulates ATM activity in response to DNA damage	(63)
miR-BART1	activates PTEN-dependent pathways including PI3K-Akt, FAK-p130 (Cas),, and Shc-MAPK/ERK1/2 signaling	(64)
miR-BART7-3p	targets human major tumor suppressor gene PTEN, regulates PI3K/Akt/GSK-3 β signal transduction	(65, 77, 83)
miR-BART10-3p	promotes Cell Proliferation and Migration by Targeting DKK1.	(66)
miR-BART10-3p/BART22	activates the canonical Wnt signaling pathway.	(69)
miR-BART11	promotes inflammation-induced carcinogenesis by targeting FOXP1.	(67, 68)
miR-BART9/BART11/BART12	downregulate Bim expression	(76)
miR-BART4-5p	regulates Bid Expression	(77)
miR-BART20-5p	targets BAD	(78)
miR-BART16	abrogates the production of IFN-stimulated genes	(79)
miR-BART22	regulates LMP2A expression	(80)
miR-BART17-5	targets Kruppel-Like Factor 2	(81, 82)
miR-BART15-3p	targets the anti-apoptotic TAX1BP1 targets NLRP3 inflammasome	(84) (85, 86)

### DNA Methylation

DNA methylation is arguably the most important mechanism in EBV-positive gastric cancer (87). Liang et al. (9) find that 216 genes were downregulated by EBV-related hypermethylation. It was also found that five signaling pathways—axon guidance, local adhesion formation, interaction between cytokines and receptors, mitogen-activated protein kinase signal transduction, and actin cytoskeleton regulation—were jointly affected by EBV-related genomic and epigenomic changes. Thus, with advances in high-throughput sequencing technology, it is possible to fully describe the mechanism of EBV-induced DNA methylation. Zhao et al. (88) displayed that the promoters of 886 genes involved in cancer-related pathways were abnormally hypermethylated in EBV-positive AGS cells, including *p14ARF*, *AQP3*, *p15*, *p16INK4A*, *DLC-1*, *p73*, *Rec8*, *ACSS1*, *WWOX*, *FAM3B*, *BCL7A*, *IHH*, *BLU*, *TRABD*, *TFF1*, *TIMP3*, *FHIT*, *DAPK*, *FSD1*, *GSTP1*, *APC*, *SSTR1*, *CRBP1*, *Mark1*, *SCRN1*, etc. (55, 89–95). Two of these genes, *PIK3CA* and *ARID1A*, presented with the highest methylation rate (96–100). Yu et al. (92) find that methylation levels of the promoter of the meiosis-specific gene, Rec8, were significantly higher in EBV-positive than EBV-negative gastric cancer tissues, and methylation levels in both these subtypes were significantly higher than that in, e.g., tissue uninfected by EBV. It is a newer tumor suppressor that is downregulated by promoter methylation in gastric cancer, especially in the EBV-related subtypes. The antitumor effect of Rec8 can be partially explained by the downregulation of cell growth–related genes (*G6PD*, *SLC2A1*, *NOL3*, *MCM2*, *SNAI1*, and *SNAI2*) and upregulation of apoptosis or migration inhibitors (*Gadd45G* and *LDHA*) and tumor inhibitors (*PinX1*, *IGFBP3*, and *ETS2*). The results suggest that methylation of the *Rec8* gene promoter is an independent risk factor for reducing the survival in patients with gastric cancer. Further, Zhao et al. (91) indicate that *SSTR1* is a newer methylated gene in gastric cancer cells in response to EBV infection, which may act as a potential tumor suppressor. Additionally, proteins expressed during the incubation period can directly affect the methylation of multiple gene promoters. For example, Wang et al. (55) find that LMP2A induces ERK phosphorylation. LMP2A also increases transcription of *DNMT3a* by activating CpG island methylation of *AQP3* promoter in EBVaGC; resulting in the loss of *AQP3* expression. Qi et al. (57) find that the overexpression of LMP1 and LMP2A inhibits COX-2 expression, mediated through the reduction of TRAF2; p-ERK aids LMP1-inhibition of COX-2 in gastric cancer. Hino et al. (61) find that LMP2A induces phosphorylation of STAT3 and activates transcription of DNMT1, leading to the CpG island methylation of *PTEN* promoter and loss of PTEN expression in EBV-related gastric cancer. Additionally, LMP2A plays an important role in epigenetic abnormalities in host gastric cells and occurrence and maintenance of EBV-related cancers (**Table 3**).

**Table 3:** Summary of methylated genes in gastric cancer.

DNA methylation****	References****
*PIK3CA*	(96–98)
*ARID1A* *Rec8* *SSTR1* *EPHB6, MDGA2, SCARF2* *AKT2, FAM3B, TGFBR1, CCNA1, MAP3K4*	(97, 99, 100) (88, 92) (88, 91) (88) (9)
*Tp73*	(90, 93)
*AQP3*	(55, 101)
*BLU, FSD1, BCL7A, Mark1*,*SCRN1, NKX3.1* *p16, FHIT, CRBP1, WWOX*,	(93)
*DLC-1*	(94)
*WIF1, NLK, APC*	(95)

### Helicobacter pylori

Simultaneous infection with EBV and *Helicobacter pylori* can occur (102). *Helicobacter pylori* and EBV infection are associated with IL-10 and IL-1RN polymorphisms (103). However, evidence for the possible interaction or antagonism of these infectious factors in carcinogenesis of gastric cancer is limited. Camargo et al. (18) compare the serological characteristics of *Helicobacter pylori* in 58 EBV-positive and 111 EBV-negative gastric cancer patients at the National Cancer Institute’s International EBV-Gastric Cancer Consortium, United States. The results suggest that the overall serum positive rate for an individual’s five immunogenic proteins is as high as 90%. Moreover, catalase antibodies were marginally associated with EBV-positive tumors. Taken together, these results suggest that infection with *Helicobacter pylori* is related to the occurrence and development of EBV-positive gastric cancer.

## What Is the Relationship Between EBV-Positive Gastric Cancer and Immunotherapy?

Statistical analysis reveals that EBV-positive gastric cancer with a low mutation burden is a subset of MSS gastric cancer and may respond to immune checkpoint therapy. In 2019, Roh et al. (104) comprehensively analyzed the status of SPC (specificity), MSI, and EBV. The results validate that the combined application of SPC, MSI, and EBV statuses could predict the efficacy and prognosis of adjuvant chemotherapy in stage II or III gastric cancer.

Gene expression profile analysis of EBVaGC patients indicates significant changes in immune response genes, which may allow recruitment of reactive immune cells to better survival outcomes in patients (105). Moreover, EBVaGC is characterized by high and low density of CD8+ T-cells and CD204+ macrophages, respectively (106, 107). Both the infiltrating immune cells and specific immune microenvironment contribute to antitumor immunity (108). However, tumor cells in EBVaGC evade immune responses via a variety of strategies. It is reported that indoleamine-pyrrole 2,3-dioxygenase (IDO1) is an effective immunosuppressive enzyme, which is upregulated in EBVaGC to resist tumor immune responses (109).

EBVaGC was found to express high levels of programmed death ligand 1 (PD-L1) in cancer and infiltrating immune cells (110). As tumor cells recruit PD-L1 to interact with programmed cell death protein 1 (PD-1) on the surface of T-cells to escape from antitumor immunity, the high expression of PD-L1 in EBVaGC can be considered to be related to tumor progression (111). Additionally, several studies have also found PD-L1 expression to be increased in patients with EBV-positive gastric cancer; patients with MSI gastric cancer showed better prognosis (112, 113).

In 2018, Kim et al. (114) reported on the molecular characterization of tissue and circulatory tumor DNA (ctDNA) in 61 patients with metastatic gastric cancer who received rescue treatment with perbrolizumab in a prospective phase 2 clinical trial. The results indicate that the objective effective rate (ORR) of perbrolizumab in the treatment of EBV-positive metastatic gastric cancer was 100%, significantly higher than the ORR of 85.7% in the treatment of metastatic microsatellite instability gastric cancer. There is a high correlation between PD-L1 positive and EBV-positive/MSI-H, suggesting that immunotherapy may be as effective in EBV-positive gastric cancer patients as it is in MSI-H patients.

In 2020, Kim et al. verified the effectiveness of immunotherapy in the treatment of EBV-positive gastric cancer (115). A total of 300 gastric cancer patients (Asian) were included in this study, of which PD-L1Cps ≥1 was positive in 178 cases (59.3%) and PD-L1Cps <1 in 122 cases (40.7%). PD-L1Cps ≥1 was significantly associated with stage I tumor (*P*=0.022), high microsatellite instability (MSI-H) (*P*<0.001), positive EBV status (*P*=0.008), and positive *Helicobacter pylori* status (*P*=0.001). In the gene expression profile, PD-L1CPs were highly positively correlated with mutation load (*P*<0.001), EBV (*P*<0.001), and microsatellite subtype (*P*<0.001). PD-L1 was expressed in 59.3% of gastric cancer patients and was associated with positive MSI and EBV status. These results suggest that patients with EBV-positive gastric cancer can benefit from immunotherapy. Research regarding the treatment of EBV-positive gastric cancer is currently under way, and we eagerly await the results.

## Discussion

**Figure 1:**
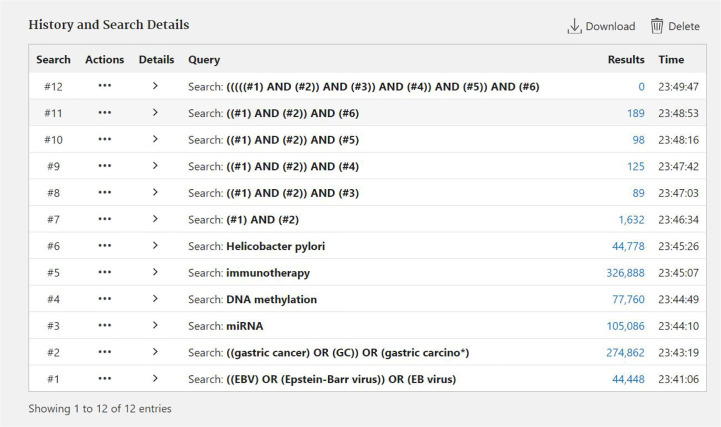
The process of searching documents by Pubmed.

**Figure 2:**
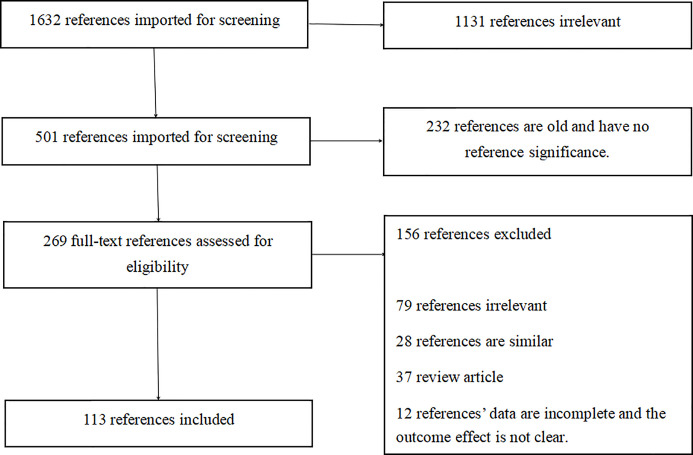
PRISMA flowchart.

This paper reviews articles dating back to the discovery of EBV-positive gastric cancer through more recent studies. We collected and read 1632 articles on Pubmed and finally selected 113 representative articles for collection (**Figures 1** and **2**). We comprehensively and systematically review EBV-positive gastric cancer, its pathological features, detection methods, pathogenesis, and potential treatment. We also describe how EBV infects the human body and affects the host’s miRNA through the expression of proteins in the latent period until it results in DNA methylation and the onset of gastric cancer. With the developments in scientific research and improvements in detection technology, our understanding of EBV-positive gastric cancer has improved. We are gradually beginning to comprehend how DNA methylation contributes to the occurrence and development of gastric cancer due to EBV infection. Moreover, recent studies have helped to understand the occurrence and development of EBV-positive gastric cancer from the perspective of gene mutations, miRNA expression, and biology. However, these details are still insufficient for improved treatment of EBV-positive gastric cancer. Although the prognosis in patients with EBV-positive gastric cancer is significantly better than other types of cancer, there is still no unified treatment regimen. On the basis of several pathogenic mechanisms, we anticipate the use of therapies that target miRNAs, DNA methylation, or immunotherapy to manage this cancer type. Furthermore, recent studies indicate that immunotherapy can help achieve complete remission in EBV-positive gastric cancer. Thus, it would be possible to decide whether to administer surgical treatment or immunotherapy in patients with early EBV-positive gastric cancer, whether anti-*Helicobacter pylori* therapy imparts significant therapeutic effects in EBV-positive gastric cancer patients, and whether these treatment modalities can also be administered in EBV-negative gastric cancer patients. Additionally, we may be able to address the issue regarding treatment of patients infected with EBV, but whose gastric cancer is not caused by EBV. In summary, we have reviewed the detection and pathogenesis of EBV-positive gastric cancer and its correlation with immunotherapy. We are in the initial stages of understanding the pathogenesis of EBV-positive gastric cancer; several unknown challenges and treatment options remain to be explored and discovered. We believe that further research and a better understanding of EBV will play a vital role in the treatment and prognosis of patients with gastric cancer.

## Search Strategy and Selection Criteria

Data for this review were identified by searching the PubMed and references from relevant articles using the search terms “EBV,” “EBV Gastric cancer,” “miRNA EBV,” “DNA methylation,” “immunotherapy,” and “*Helicobacter pylori*.” Abstracts and reports from meetings were included only when they related directly to previously published work. Only articles published in English between 1987 and 2020 were included (**Figures 1** and **2**).

## Author Contributions

XC and HLv conceived the article and decided the theme of the manuscript. YW, HLei, and S-QW reviewed published articles and materials and integrated them. KS and KJ analyzed the materials and drafted the manuscript. All authors contributed to the article and approved the submitted version.

## Funding 

This study received funding from the National Natural Science Foundation of China (81472714); and Joint Co-construction Project of Medical Science and Technique Foundation Plan of Henan Province (2018020486) 1000 Talents Program of Central Plains of Henan Province (204200510023).

## Conflict of Interest

The authors declare that the research was conducted in the absence of any commercial or financial relationships that could be construed as a potential conflict of interest.
